# Weight stigma speaks Italian, too

**DOI:** 10.1007/s40618-022-01971-8

**Published:** 2022-11-28

**Authors:** G. Muscogiuri, L. Barrea, L. Verde, A. Docimo, S. Savastano, D. Di Pauli, A. Colao

**Affiliations:** 1 Dipartimento Di Medicina Clinica E Chirurgia, Unit of Endocrinology, Federico II University Medical School of Naples, Via Sergio Pansini 5, 80131 Naples, Italy; 2 Department of Clinical Medicine and Surgery, Endocrinology Unit, Centro Italiano Per La Cura E Il Benessere del Paziente Con Obesità (C.I.B.O), University Medical School of Naples, Via Sergio Pansini 5, 80131 Naples, Italy; 3 Cattedra Unesco “Educazione Alla Salute E Allo Sviluppo Sostenibile”, University Federico II, Naples, Italy; 4 Dipartimento Di Scienze Umanistiche, Università Telematica Pegaso, Via Porzio, Centro Direzionale, Isola F2, 80143 Naples, Italy; 5 Independent Researcher, Verona, Italy

**Keywords:** Obesity, Weight stigma, Diet, Psychological health, Childhood obesity, Obesity questionnaire

## Abstract

**Purpose:**

Weight stigma is the negative weight related attitudes and beliefs towards individuals because of their overweight or obesity. Subjects with obesity are often victim of weight-related stigma resulting in a significant negative social consequence. As obesity epidemic is growing so fast, there is urgency to act on weight-stigma related social consequences being potentially serious and pervasive. This study investigated experiences, interpersonal sources, and context of weight stigma in Italy in a sample of adult subjects with obesity.

**Methods:**

An online questionnaire was distributed to respondents via a snowball sampling method among subjects with obesity belonging to Italian Associations for people living with obesity aged 18 years and above.

**Results:**

Four hundred and three respondents (47.18 ± 9.44 years; body mass index (BMI) 33.2 ± 8.48 kg/m^2^) participated to the study. Most respondents were females (94.8%). The age first dieted was 15.82 ± 7.12 years.

The mean period of obesity was 27.49 ± 11.41 years. Frequency analyses reported that stigmatizing situations were experienced by 98% of participants: 94.82% during adulthood, 89.88% during adolescence and 75.39% during childhood. Verbal mistreatments (92.43%) was the most reported stigmatizing situation, strangers (92.43%) were the most common interpersonal sources of stigma and public settings (88.08%) were the most common location of stigma.

**Conclusions:**

Identifying strategies acting on the identified weight stigma targets could contribute to reduce weight stigma and thus to result in important implications for obesity treatment in Italy.

## Introduction

Weight stigma is the negative weight related attitudes and beliefs including stereotypes, rejection, and prejudice towards individuals because of their overweight or obesity [1]. Subjects with obesity are often victim of weight-related stigma resulting in a significant negative social consequence [2]. Weight stigma experiences are very common in subjects with obesity [3] that in turn internalize them blaming and criticizing themselves as the only cause of their weight condition [4].

Previous studies showed that weight stigma is worldwide and over 50% of adults surveyed across six different countries (Australia, Canada, France, Germany, the UK, and the US) report experiencing weight stigma [5] but there are missing information regarding “the weight” of weight stigma in Italy.

As obesity epidemic is growing so fast [6], there is urgency to act on weight-stigma related social consequences since these could potentially be serious and pervasive leading to unfair treatment, prejudice and even discrimination. In addition, weight stigma could create a fertile ground for the onset of depression, body images distress, psychiatric symptoms, and decreased self-acceptance [7–9]. The negative consequences for physical health include unhealthy eating patterns (binge eating and increased food consumption), avoidance of physical activity, and less use of health care [5, 10–12]. Weight-related stigma includes a wide variety of stages, going from repeated mocking and mobbing to harassment and hostility [13, 14]. In addition, people affected by obesity report experiencing undesired attention, social rejection, and discrimination [15].

People from any age can suffer from this stigma. Indeed, the anti-fat attitudes has been reported to begin early in the childhood as young as preschool age [16]. In a study carried out in 2016 adolescents, weight-based teasing has been associated to binge eating at a 5 years of follow up in both males and females and even after adjustment for confounding factors such as age, race/ethnicity and socioeconomic status [17]. Weight stigma has been reported in educational settings towards students with obesity by peers, classmate, teachers and school administrators [18]. It has also been detected in healthcare environments, where patients with obesity are subjected by bias by healthcare professionals including those specialized in obesity [19–24] and in workplace settings where overweight employees are victim of negative judgements by co-workers and supervisors [25, 26].

A gender difference in weight-stigma related effects also emerged: indeed, weight teasing results in unhealthy weight control behaviors among men and frequent dieting in women [12].

Although increasing attention on weight stigma because of its important repercussions on the management of obesity and overall quality of life, there is a need of epidemiologic studies in order to quantify this phenomenon and to set up a strategy to act.

Thus, the objective of the present study was to carry out a survey in a large sample of Italian adults with obesity by documenting and examining sources of weight bias, life domains and locations where it occurs.

## Materials and methods

A snowball sampling method was used to spread an online questionnaire, on the Google Form platform, between subjects affected by obesity who belonged to Italian Associations for people living with obesity. The minimum age required was 18 years old and the maximum age was 71 years old. The BMI range was between 18.7 and 59.9 kg/m^2^. It was also asked to the subjects to involve the people they know, inviting them to answer the questionnaire too. In this cross-sectional study all search procedures were carried out in accordance with the pertinent guidelines and regulations of the Declaration of Helsinki. All respondents signed a valid informed consent.

### Questionnaire on weight stigma

The questionnaire was developed through a literature review [1, 12]. Questions from 1 to 7 covered sociodemographic and anthropometric characteristics. Participants were asked to report their age (years), gender, height (cm), weight (kg), childhood weight status (using “underweight”, “normal weight” or “overweight” as response choices), age of first dieting attempt and how many years they have been suffering from obesity. Questions from 8 to 11 dealt with the current therapeutic management of obesity (using “nutritional”, “pharmacological” and “psychological” as response choices) and any previous or planned bariatric surgery. Questions from 12 to 45 covered ‘type of stigma’ (verbal, physical barriers, being avoided, excluded, ignored, job discrimination, being attacked), ‘context of stigma’ (home, public place, school, work, medical facility, mode of transportation, sports facility) and ‘source of stigma’ (men, women, children, adolescent, adult, peer/friends, parent, sibling, boyfriend/girlfriend, spouse, stranger, other family member, health professional, nurse, boss/supervisor, sales clerk/server, teacher/professor, administrative staff). The available answers were never, rarely, occasionally, often, always. Question 46 was an open-ended question: “Can you describe where, when and by whom you suffered what you consider to be the worst experience of stigma experienced because of your weight?”. A pilot assessment was conducted among the first 30 respondents recruited through snowball sampling to ensure the questions were clearly written, easily understood and unambiguous.

### Statistical analysis

Results have been described as mean ± (standard deviation) SD or number (percentage). Differences in multiple groups were analyzed by ANOVA test followed by the Bonferroni post-hoc test. SPSS software (PASW version 21.0, SPSS Inc., Chicago, IL, USA) and the MedCalc® package (version 12.3.0 1993–2012 MedCalc Software bvba-MedCalc Software, Mariakerke, Belgium) were used to analyze the collected data.

## Results

A total of 403 respondents (47.18 ± 9.44 years; BMI: 33.2 ± 8.48 kg/m^2^) participated to the study. Most respondents were females (94.8%). The age first dieted was 15.82 ± 7.12 years. With respects to childhood weight, 32.75% had normal weight, 61.46% was overweight and 5.79% was underweight. The mean period of obesity was 27.49 ± 11.41 years. Sixty % of subjects reported to be on treatment for weight excess: 71.18% underwent to bariatric surgery, 8.47% were taking anti-obesity drugs, 5.51% was candidate for bariatric surgery, 14.4% were following psychological and 77.12% nutritional treatments.

Regarding subjects that underwent to bariatric surgery, 7.77% underwent to gastric banding, 45.95% to sleeve gastrectomy, 11.82% to gastric by-pass, 15.54% to mini-gastric by-pass, 6.76% to Roux-en-Y gastric bypass, 1.69% to intragastric balloon and 1.35% to other types of surgery, whilst 9.12% of subjects underwent to more than one bariatric surgery procedure.

As expected, subjects that underwent to bariatric procedures had significantly lower BMI (30.52 ± 6.36 kg/m^2^) than subjects that did not (BMI 38.81 ± 9.73 kg/m^2^) and then subjects candidate for bariatric surgery (BMI 43.25 ± 7.29 kg/m^2^) (*p*  <  0.001). Subjects that underwent to intragastric balloon had significantly higher BMI (45.3 ± 4.3 kg/m^2^) than other groups of subjects underwent to other bariatric surgery procedures (BMI 34.4 ± 6.4 kg/m^2^ gastric banding, 31.2 ± 7.5 kg/m^2^ sleeve gastrectomy, 29.8 ± 6.1 kg/m^2^ gastric bypass, 30.2 ± 7.1 kg/m^2^ mini-gastric bypass, 30.5 ± 5.7 kg/m^2^ Roux-en-Y gastric bypass) (*p* = 0.003). Subjects that underwent to more than one bariatric surgery procedure were still in a weight-excess state (BMI 31.32 ± 6.41 kg/m^2^).

The answers to the qualitative question (“Can you describe where, when and by whom you suffered what you consider to be the worst experience of stigma experienced because of your weight?”) showed that the worst stigma experiences were very variable in terms of settings and individuals. The majority of the subjects reported their worst stigma experience occurred in adulthood and were enacted by another adult. Selected examples of response are reported below:

“When I was trying to get my gynecologist to understand that I was having strong contractions in the seventh month of pregnancy, she told me that it was just my body being tired because of too much weight, whereas it was pre-eclampsia. She didn't even examine me, just a phone interview because the only problem with my pregnancy for her was the weight”

“At the bank I was stuck between the security doors. The automated voice said: 'Enter one person at a time'. The vigilante watched me go in and out, snickering. A terrible humiliation!”

“At school, a teacher explaining how the scales worked made a joke about my weight in front of everyone… they all laughed out loud, especially him.”

## Experiences of stigma

Frequency analyses reported that stigmatizing situations were experienced by 98% of participants: 94.82% during adulthood, 89.88% during adolescence and 75.39% during childhood.

### Stigmatizing situations

**Fig. 1:**
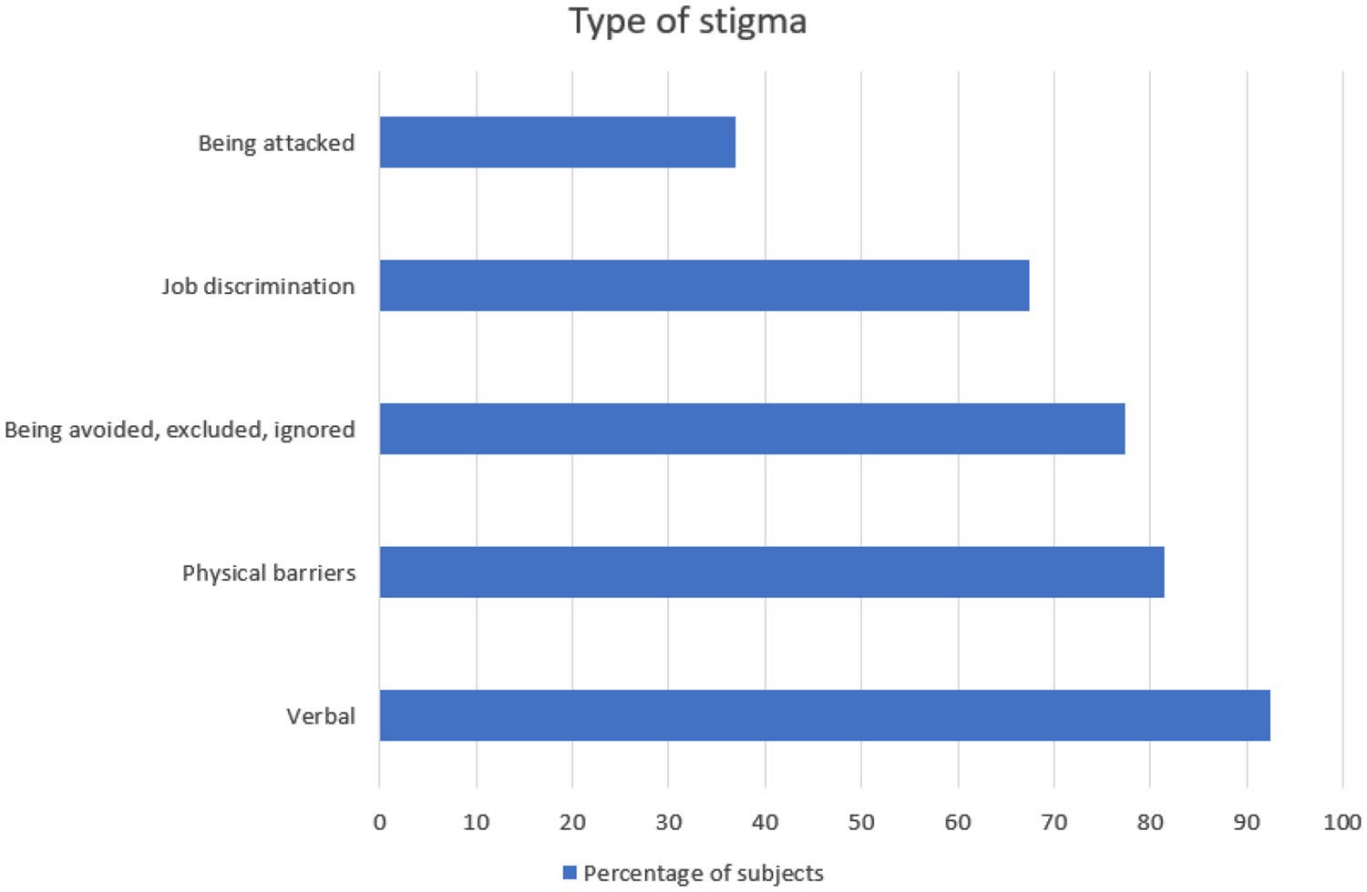
Type of stigma in the study population

Regarding stigmatizing situations, we found that verbal mistreatments was the most common: indeed, 92.43% of respondents experienced nasty comments from children, family members and strangers. The 81.36% of the participants reported to have experienced physical barriers and obstacles, whilst the 77.45% of them felt to be avoided, excluded, or ignored. Job discrimination was referred from the 67.42% of the subjects and 37% of them were attacked (Fig. 1).

Table 1 presents descriptive statistics on the stigma subscales.

**Table 1:** Type of stigma in the study population

Type of stigma	Never	Rarely	Occasionally	Often	Always
Verbal	29 (7.6)	65 (17.0)	85 (22.2)	146 (38.1)	58 (15.1)
Physical barriers	71 (18.6)	55 (14.4)	64 (16.8)	126 (33.07)	65 (17.1)
Being avoided, excluded, or ignored	83 (22.6)	80 (21.7)	84 (22.8)	95 (25.8)	26 (7.1)
Job discrimination	115 (32.6)	65 (18.4)	73 (20.7)	73 (20.7)	27 (7.7)
Being attacked	218 (63.0)	60 (17.3)	39 (9.8)	21 (6.1)	8 (2.3)

Data are expressed as *n* (%).

### Interpersonal sources of stigma

**Fig. 2:**
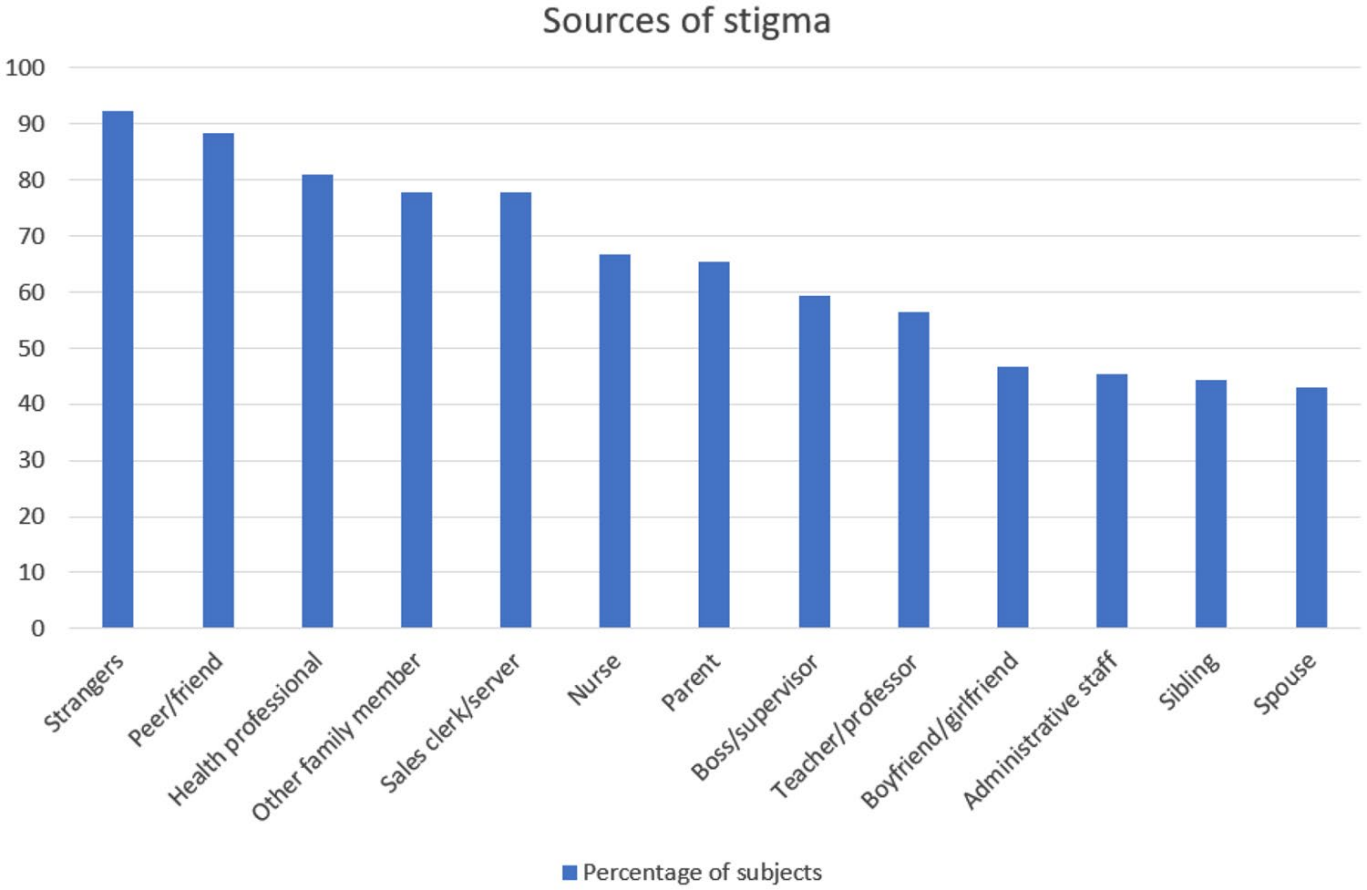
Interpersonal sources of stigma in the study population

Ninety-four % of respondents were stigmatized by men and 95% by women. The most common and frequently reported sources of stigma were adults (95.6%) followed by adolescents (88.28%) and children (81.44%). The most reported interpersonal source of stigma were strangers (92.43%). The respondents also reported to be stigmatized by peer/friends (88.28%), health professionals (80.9%), family members (77.84%), sales clerks/servers (77.75%), nurses (66.75%), parents (65.27%), boss/supervisors (59.31%), teachers/professors (56.43%), boyfriend/girlfriend (46.06%), administrative staff (45.4%), siblings (44.19%), and spouse (42.86%) (Fig. 2).

Table 2 report descriptive statistics of interpersonal sources of stigma.

**Table 2:** Interpersonal sources of stigma in the study population

Interpersonal sources of stigma	Never	Rarely	Occasionally	Often	Always
Gender of perpetrator					
Men	23 (6.1)	74 (19.6)	120 (31.8)	129 (34.1)	32 (8.5)
Women	19 (4.9)	58 (15.3)	140 (36.3)	141 (36.5)	28 (7.3)
Age of perpetrator					
Adult	17 (4.4)	79 (20.5)	121 (31.4)	140 (36.3)	29 (7.5)
Adolescent	43 (11.7)	59 (16.1)	99 (27.0)	136 (37.1)	30 (8.2)
Children	67 (18.6)	88 (24.4)	98 (27.1)	93 (25.8)	15 (4.2)
Source of stigma					
Strangers	28 (7.6)	66 (17.8)	104 (28.1)	131 (35.4)	41 (11.1)
Peer/friends	43 (11.7)	93 (25.3)	90 (24.6)	122 (33.2)	19 (5.2)
Health professional	72 (19.1)	78, (20.7)	87, (23.1)	110, (29.2)	30, (8.0)
Other family member	80 (22.2)	99 (27.4)	93 (25.8)	69 (19.1)	20 (5.5)
Sales clerk/server	79 (22.3)	63 (17.8)	96 (27.0)	86 (24.2)	31 (8.7)
Nurse	118 (33.2)	75, (21.1)	75, (21.1)	69, (19.4)	18 (5.1)
Parent	124 (34.7)	76 (21.3)	64 (17.9)	69 (19.3)	24 (6.7)
Boss/supervisor	142 (40.7)	81 (23.2)	74 (21.2)	37 (10.6)	15 (4.3)
Teacher/professor	149 (43.6)	72 (21.1)	72 (21.1)	41 (12.0)	8 (2.3)
Boyfriend/girlfriend	185 (54.0)	77 (22.5)	47 (13.7)	28 (8.2)	6 (1.8)
Administrative staff	190 (54.6)	81 (23.3)	46 (13.2)	25 (7.2)	6 (1.7)
Sibling	192 (55.8)	58 (16.9)	50 (14.5)	31 (9.0)	13 (3.8)
Spouse	192 (57.1)	74 (22.0)	36 (10.7)	24 (7.1)	10 (83.0)

Data are expressed as *n* (%)

### Context of stigma

**Fig. 3:**
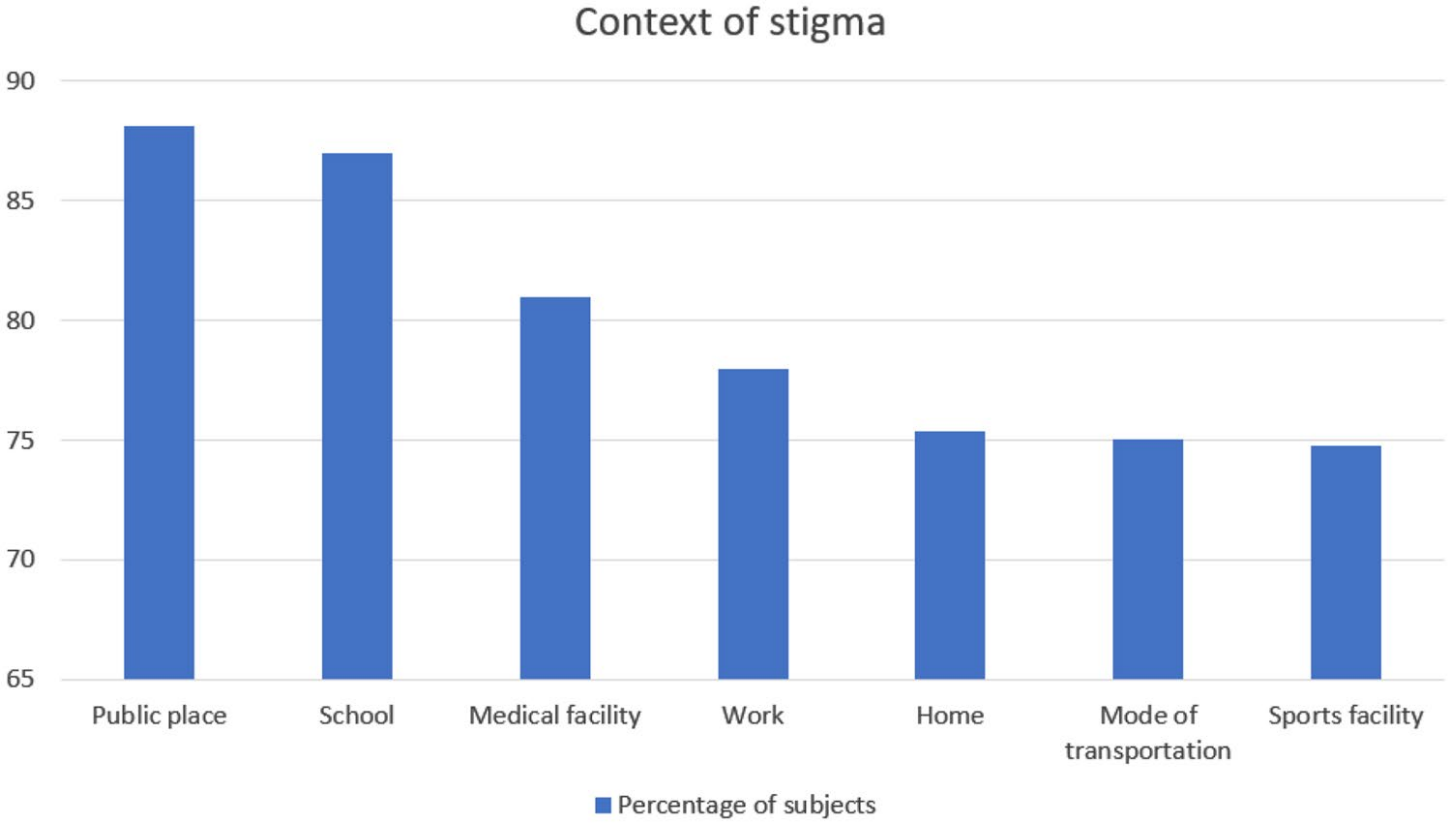
Context of stigma in the study population

Seventy-five % of the respondents reported public places (88.08%) as the most common location of stigma followed by school (86.94%), medical facility (80.93%), work (77.97%), home (75.35%), mode of transportation (75%) and sports facilities (74.72%) (Fig. 3).

Table 3 report descriptive statistics of context of stigma.

**Table 3:** Context of stigma in the study population

Context of stigma	Never	Rarely	Occasionally	Often	Always
Public place	44 (11.9)	70 (19.0)	140 (37.9)	104 (28.2)	11 (3.0)
School	47 (13.1)	53 (14.7)	93 (25.8)	126 (35.0)	41 (11.4)
Medical facility	70 (19.1)	78 (21.3)	113 (30.8)	87 (23.7)	19 (5.2)
Work	78 (22.0)	86 (24.3)	121 (31.2)	57 (16.1)	12 (3.4)
Home	88 (24.7)	116 (32.5)	75 (21.0)	58 (16.3)	20 (5.6)
Mode of transportation	88 (25.0)	76 (21.6)	105 (29.8)	74 (21.0)	9 (2.6)
Sports facility	90 (25.3)	74 (20.8)	96 (27.0)	77 (21.6)	19 (5.3)

Data are expressed as *n* (%)

## Discussion

Being a target of weight stigma in a variety of forms and occasions has been reported by participants in this study. Most respondents reported to be victim of stigmatizing situations during adulthood. In particular, the most common types of weight stigma reported was verbal mistreatments experiencing nasty comments from children, family members and strangers. This finding has been previously reported by Puhl et al. that investigated experiences of weight stigma, sources of stigma, coping strategies and psychological functioning and eating behaviors in a sample of 2671 subjects with overweight or obesity [12]. In agreement with our finding, they found that the most common stigmatizing situation reported verbal mistreatments, i.e., others making negative assumptions, receiving nasty comments from children, encountering inappropriate comments from doctors and receiving negative comments from family members [12].

Participants reported being stigmatized by a variety of interpersonal sources, the most frequent being strangers followed by friends, health professionals, sales clerks/servers at stores, family members, nurses, parents, boss/supervisors, teachers/professors, administrative staff, sibling, boyfriend/girlfriend and spouse, thus suggesting that stigma reduction strategies need to target a range of individuals in multiple settings. Our results are consistent with previous research that identify strangers as the most common interpersonal source of weight stigma [27–29].

Indeed, Falkner et al. found that the most reported sources of mistreatment among 800 women enrolled in a weight gain prevention were strangers followed by spouse or loved one [27]. Similarly, Himmelstein et al. reported that the most common sources of weight stigma in 1513 men were strangers followed by peers and family members [28]. This is broadly consistent with a study in 46 community man and women who took part in an ecological momentary assessment study of their experiences with weight stigma that found that stigma was perpetrated by a variety of sources and in several different settings but mostly occurred by strangers [29].

An interesting finding of our study was that doctors, that should be immune to weight bias, were not and conversely, they were referred as ones of the most frequent source of stigma [30, 31].

This is in accord with a previous research carried out by Schwartz et al., who studied 389 health professionals attending the opening session of an international obesity conference [30]. The Implicit Associations Test (IAT) was used to assess the overall implicit attitude to weight bias (through the automatic memory-based associations of “people with obesity” and “thin people” with “good” vs. “bad”). Then, 3 ranges of stereotypes were identified: lazy-motivated, smart-stupid, and valuable-worthless.

Interestingly, in health professional, a significant pro-thin and anti-fat implicit bias was highlighted on the IAT, and the subjects endorsed significantly the implicit stereotypes of lazy, stupid, and worthless [30]. Similarly, Teachman and Brownell found evidence for implicit anti-fat bias for both the attitude and stereotype measures (evaluated also in this case with the IAT) in 84 health professionals who treated obesity [31].

Some strategies have been demonstrated to help reducing the weight stigma between the health professionals, such as developing dedicated educational programs and giving information about obesity determinants, which often are not dependent on the patient’s will [32]. In addition, it has to be considered that not always the weight loss is achievable and safe for all the subjects with an increased weight [32].

Finally, as previously reported [29, 33], we found that weight stigma occurred most in public places. Vartanian et al. carried out an ecological momentary assessment study of their experiences with weight stigma in 46 community adults finding that weight stigma occurred frequently in public places as well as at home [29]. In agreement with this evidence, Hatzenbuehler et al. carried out a study in 22 231 individuals with overweight or obesity from Wave 2 of the National Epidemiologic Survey on Alcohol and Related Conditions (NESARC) (a cross-sectional nationally representative study of noninstitutionalized US adults) study, to investigate the associations between perceived weight discrimination and the prevalence of psychiatric disorders [33]. Results showed that perceived weight discrimination is most likely to be experienced in public, followed by insurance and health care settings [33].

In conclusion our study is the first to investigate the weight stigma in Italian adults with obesity by documenting and examining sources of weight bias, life domains and locations where it occurs. In the studied population verbal mistreatments was the most reported stigmatizing situation, strangers were the most common interpersonal sources of stigma and public settings were the most common location of stigma. Even if the population sample is relatively small and the data may be impacted by small biases, as they are self-reported, this study has demonstrated the impact of the wight stigma on the patients. Identifying strategies acting on these targets could contribute to reduce weight stigma and thus to improve the management of obesity in Italy.

## Data Availability

The datasets generated during and/or analyzed during the current study are available from the corresponding author on reasonable request.
